# NIHR Race Equality Framework: development of a tool for addressing racial equality in public involvement

**DOI:** 10.1186/s40900-024-00569-z

**Published:** 2024-05-07

**Authors:** David Faluyi, Pavel V. Ovseiko, Krysia Dziedzic, Fay Scott, on behalf of the NIHR Race Equality Public Action Group, André Tulloch, Caroline Barker, Claire Wallace-Watson, Jon Cole, John Castledine, Kate Holmes, Katie Cook, Laurie Oliva, Mark Slocombe, Mike Rogers, Nikki Bent, Pavel Ovseiko, Royston John, Sandra Richards, Sarah Knowles, Zahra Kosar

**Affiliations:** 1 King’s College Hospital NHS Foundation Trust, Denmark Hill, London, SE5 9RS UK; 2 Radcliffe Department of Medicine, University of Oxford, John Radcliffe Hospital, Oxford, OX3 9DU UK; 3 Impact Accelerator Unit, School of Medicine, Keele University, Keele, ST5 5BG UK; 4 National Insititute for Health and Care Research (NIHR) Coordinating Centre, Grange House, 15 Church Street, Twickenham, TW1 3NL UK; 5 NIHR Race Equality Public Action Group, NIHR Coordinating Centre, Grange House, 15 Church Street, Twickenham, TW1 3NL UK

**Keywords:** Race equality, Antiracism, Inequality, Diversity, Inclusion, Public involvement, Public engagement, Health research, NIHR

## Abstract

**Background:**

While there has been a long recognition of the importance of race equality in health and care research, there is a lack of sustained action among research funding and research performing organisations to address racial equality in public involvement. This paper describes how the UK’s National Institute for Health and Care Research (NIHR) convened a Race Equality Public Action Group (REPAG), which co-developed with public contributors and stakeholders a Race Equality Framework – a tool for addressing racial equality in public involvement.

**Methods:**

The REPAG, through meetings and discussions, defined the focus of the Framework, and developed an initial draft of the Framework. Public contributors identified the need for broader consultation with other public members. Three community consultation events with a total of 59 members of Black African-, Asian- and Caribbean-heritage communities were held to seek their views on health and care research generally and on the draft Framework specifically. The draft Framework was modified and piloted among 16 organisations delivering health and care research. Following feedback from the pilot, the Framework was modified and prepared for publication.

**Results:**

The Framework is designed as a self-assessment tool comprised of 50 questions pertaining to five domains of organisational activity: 1) individual responsibility, 2) leadership, 3) public partnerships, 4) recruitment, and 5) systems and processes. The questions were co-designed with REPAG public members and provide key concepts and elements of good practice that organisations should consider and address on their path to achieving racial competence. The accompanying materials provide implementation guidance with 20 detailed steps, case studies of actions taken in seven pilot organisations, and links to additional resources. The pilot demonstrated the feasibility of conducting a meaningful self-assessment over a period of three months and the usefulness of the results for developing longer-term action plans.

**Conclusion:**

The Framework represents the first self-assessment tool for addressing racial equality in public involvement. Co-design with REPAG public members enhanced its authenticity and practicality. Organisations in the field of health and care research and any other organisations that use partnerships with the public are encouraged to adopt the Framework.

**Supplementary Information:**

The online version contains supplementary material available at 10.1186/s40900-024-00569-z.

## Abstract (plain language summary)

For a long time, race equality within research has not been a priority. To address this the NIHR created a group to come up with solutions to tackle this – the Race Equality Public Action Group. Recognising the need to help research organisations with how they involved members of the public from diverse groups, the Race Equality Framework was born. The Race Equality Framework was devised to help organisations become more inclusive, develop better links with diverse communities and make their work more equitable.

Consultation with members of the public from Black African-, Asian- and Caribbean-heritage communities were held and were essential in developing the Framework. Sixteen organisations who are involved in health and care research were recruited as pilot partners. They worked through the Framework and provided further feedback and input.

The final product is a self-assessment tool which comprises of 50 questions across five domains. These domains are: 1) individual responsibility, 2) leadership, 3) public partnerships, 4) recruitment, and 5) systems and processes. The questions are designed to promote reflection and improve organisational good practice.

This is the first tool of its kind, and we recommend its use for health and care research organisations that work closely and develop partnerships with members of the public.

**Supplementary Information:**

The online version contains supplementary material available at 10.1186/s40900-024-00569-z.

## Background

There has for a long time been a recognition that racial and ethnic minorities often have poorer health outcomes but remain under-represented in health and care research [1–7]. The COVID-19 pandemic has further highlighted the importance of racial and ethnic equality in health and care research [8]. While racial and ethnic minorities were disproportionally affected by the pandemic [9–11], they were under-represented in clinical trials as research participants [12, 13]. In response, there have been renewed efforts to adequately represent racial and ethnic minorities in research as research participants [14–17].

Yet, there remains an urgent need for improving the public involvement and engagement of racial and ethnic minorities in all aspects of research beyond being just research participants, including in identifying research priorities, setting research questions, shaping study design, being co-applicants, and actively informing all stages of research and dissemination [18–20]. Public involvement in research means that members of the public are actively involved in research projects and in research organisations to the effect that “research being carried out ‘with’ or ‘by’ members of the public rather than ‘to’, ‘about’ or ‘for’ them” [21]. Public members can offer lived experience (expertise by experience).

The National Institute for Health and Care Research (NIHR) is the UK’s largest public health and care research funding organisation funded by tax-payers through the Department of Health and Social Care “to improve the health and wealth of the nation through research” [22]. Noting that within the UK context, care research generates evidence to improve, expand and strengthen the way social care is provided for users of care services, carers, the social care workforce, and the public. The NIHR places public involvement at the centre of its activities and provides research performing organisations and researchers with dedicated funding for public involvement. The NIHR requires all funding applications to demonstrate how members of the public were involved in developing research proposals and how they will continue to be involved in informing research studies [23].

In line with the UK Standards for Public Involvement, NIHR-funded research is required to be “informed by a diversity of public experience and insight” [24]. However, a survey of NIHR public contributors conducted between December 2018 and January 2019 showed a lack of racial and ethnic diversity in public involvement [25]. While Asian and Black public contributors represented 3% and 2% of the survey respondents respectively [25], Asian and Black minorities were estimated to account for 8% and 3.5% of the general population in England and Wales in 2019, respectively [26]. In April 2020, the NIHR launched a new Centre for Engagement and Dissemination that brought together its activities in patient and public involvement, engagement and participation with its strengths in research dissemination.

On behalf of the NIHR, in October 2020, FS founded a Race Equality Public Action Group (REPAG) – a proactive public partnership response to the emerging findings from public health reports of longstanding inequalities exacerbated by COVID-19 [10]. FS built the evidence-based case, and through iterative conversations with senior organisational stakeholders gained sponsorship for the Group. FS then recruited REPAG members, who included a diverse group of public contributors with lived experience of race inequality, together with NIHR academic partners and NIHR staff to co-develop an actionable improvement plan [27]. As part of the wider NIHR research inclusion strategy [28] being formulated and developed by senior leadership from across the NIHR, the REPAG was given the remit to focus on actions that relate directly to public and patient involvement and engagement in the following two broad strategic areas being considered by the NIHR Research Inclusion programme: 1) involving in research, patients, carers and members of the public who reflect the diversity of our society; and 2) focusing on those most affected by health and care challenges.

Within this broader societal and organisational context, the REPAG’s distinct purpose was defined as follows [29]:

- To help the NIHR strengthen its understanding of race inequality in health and social care research;
- To advise on actions for the NIHR to take to ensure that race equality is embedded in interactions between the research community and patients, service users, carers and the public;
- To prioritise the voices of those most affected by health and care challenges.

Seeking to build on the broader standard ‘inclusive opportunities’ in the UK standards of public involvement in health and care research [24], the REPAG decided to co-develop with public contributors and stakeholders the NIHR Race Equality Framework. This is a self-assessment tool designed to help organisations address racial equality in public involvement by becoming more inclusive, developing better links with diverse communities, and making their work more equitable, building on the broader theme of inclusive opportunities in the UK standards for public involvement. Below, we describe the process that was used by the REPAG to develop and launch the Framework.

## Methods

The overall underpinning approach for the REPAG became Knowledge Mobilisation, i.e. taking knowledge from different places and transforming it into innovations that can be used by those who need it. Importantly, knowledge is multi-directional and can flow between stakeholders.

Knowledge Mobilisation is supported by a number of models and theories that help support the planning, understanding and evaluation of new innovations such as the Race Equality Framework into practice [30]. Whilst many of these have not been developed with public involvement, or address the needs of the public in Knowledge Mobilisation, Communities of Practice have been successfully used in public involvement [31].

A Community of Practice can be considered ‘a group of people, who share a concern, a set of problems, or a passion about a topic, and who deepen their knowledge and expertise in this area by interacting on an ongoing basis’ [32]. The REPAG considered itself as a Community of Practice and used the following recommendations to optimise the process of knowledge mobilisation [31]:

- creating the best environment for knowledge exchange
- creating an equitable platform that allows everyone to participate
- supporting members to make sense of the Community of Practice in a flexible way

In line with such recommendations, REPAG firstly established the infrastructure to provide administrative and wider support to the group and to facilitate meetings. This included the core team with funding, organisational, technical and digital expertise, project management, and regular communication (announcements, news sharing) and offered more formal roles to public members such as initiating the Race Equality Framework concept.

REPAG member selection for co-production activities gave a sense of ownership over the Race Equality Framework. REPAG also acknowledged the iterative nature of the process and that the group aims, and purpose can evolve over time. REPAG considered the varied skill set, knowledge base and expertise that all members offered and facilitated relationship building and co-production, e.g. by creating a culture of trust, confidence, and a safe space for deliberations.

## Development of the framework

Between October 2020 and April 2022, REPAG members held monthly meetings, typically two hours long, and facilitated most of the time online via Zoom, with both plenary and break out group sessions. In order to provide a strong foundation for this work by preparing REPAG members for honest and reflective conversations, FS recruited an external trainer with significant experience in this area. With NIHR occupying a strategic position within the health and care research landscape, the group discussed recommendations it could disseminate to research active organisations that it funds, that they could address the documented barriers to:

- Meaningful involvement of public contributors from communities under-served by research [17];
- Co-production and testing of interventions shaped to address different support needs and tackle known disparities in health and care outcomes [33], in line with UK public sector equality duties [34].

**Fig. 1:**
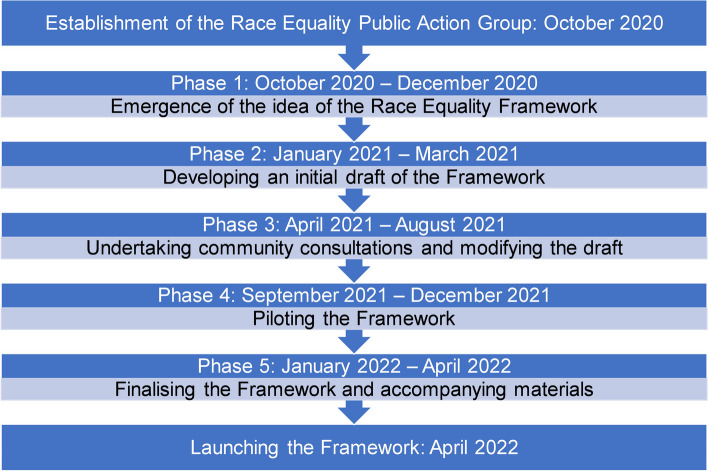
Development of the NIHR Race Equality Framework

Ideas and suggestions were captured and aggregated using Jamboard, a digital interactive whiteboard, as well as via email. Key to the success of these meetings was the conscious steps taken and efforts made to ensure trust, confidence, a safe space for REPAG deliberations, and equal partnerships with the REPAG public contributors. A flow diagram (Fig. 1) outlines the major phases of work.

A full list of the 19 people who have made a substantial contribution to the Framework in one or more phases is given in the acknowledgements. They were past and present REPAG members representing the NIHR (9), NIHR and REPAG public contributors (6), and academic partners (4). REPAG public contributors were recruited from existing public involvement networks known to the NIHR Senior Public Involvement Manager and REPAG Founder and Co-Chair (FS).

### Phase 1: emergence of the idea of the Framework (October 2020 – December 2020)

A series of conscious actions were taken to develop an equitable platform that allows everyone to participate in knowledge exchange with trust, confidence, and a safe space for deliberations within REPAG. REPAG was co-chaired by a senior public contributor and a member of NIHR. In the first two meetings, REPAG members were introduced to each other and the wider context for the group, including the academic literature and government reports cited in the ‘Background’ section above, its purpose and aims, and carried out visioning exercises around key changes we should foster and our role within that. The tone of the meetings was welcoming and respectful of confidences being shared from life experiences that spanned a range of intersectionality with race, including gender, ethnicity, age, career history, health, disability and sexuality. The public Co-Chair (RJ) actively encouraged people to grow to be comfortable with being uncomfortable sharing critical insights and reflections. This surfaced the strong ambition to be action orientated, and for public involvement to move beyond the passive representation to active involvement where the different community needs are valued and acted on to shape and inform research to increase the likelihood of realising health benefits in communities under-served by research.

To further create the best environment for knowledge exchange within REPAG, two specific approaches were taken. Firstly, REPAG members took part in a bespoke series of three race equality learning and development sessions. These interactive sessions were co-developed by the public Co-Chair of REPAG (RJ) and an external trainer, both of which have considerable experience in leading personal and professional development courses. In these training sessions, REPAG members explored social identities, intersectionality, and lived experiences of barriers and facilitators to inclusion. The second approach was to be unequivocally action orientated. This was achieved by working at pace to co-develop the REPAG strategic action plan for year one and beyond, and related internal NIHR briefing papers, through discussion sessions across the first four monthly meetings and collaborative writing up of the agreed ideas in small working groups with a cross section of members between meetings. REPAG members shared their experiences and insights in internal NIHR-wide REPAG-hosted events such as ‘Black men, well-being and toxic corridors’ and ‘Allyship in action’. The events proved to be insightful and thought provoking and generated follow up discussions with colleagues on the practical and personal actions to support positive cultural change.

Governance procedures were put in place which included the minuting of actions arising in the meetings, with responsive delivery of actions against shown in subsequent meetings. In addition, fairness was demonstrated by proactive management of public member recognition fees, which were adjusted in line with the NIHR policy [35] and complexity of the tasks.

The idea for the Framework emerged during online meetings and deliberations of the REPAG strategic action plan in October 2020 – December 2020. The initial idea itself was suggested by DF (REPAG public contributor). Key discussions amongst REPAG members concerned the desire to challenge and change systemic barriers to the under-representation of racialised communities in public involvement and establish ways to develop racial competence in research performing organisations. The definition of racial competence was proposed by FS and after reflection and deliberation approved by all REPAG members [36]:

Racial competence is the ability to recognise and check one's own bias; interact with racial diversity in a positive manner; and have open and honest conversations about race in ways that show a willingness to hear, learn and take action. Racial competence means understanding the impact of structural racism and fostering a culture of allyship that challenges organisational practices and behaviours that exclude Black African-, Asian and Caribbean-heritage people and other racialised groups. Being racially competent means translating our statements into action to promote equity of voice and equality of opportunity.

In November and December 2020, discussions were held with public members, facilitated by the public Co-Chair (RJ), on moving away from the controversial acronym BAME (Black, Asian, and Minority Ethnic), which alienated communities and caused hurt. It went on to propose alternative wording that better reflects the histories, identities, and cultures of those communities. The group has adopted the new term *Black African-, Asian- and Caribbean-heritage people and other racialised groups*.

The definitions of race and equality were adopted from the Equality and Human Rights Commission [37]:

A race is a group of people defined by their colour, nationality (including citizenship) ethnicity or national origins.

Equality is about ensuring that every individual has an equal opportunity to make the most of their lives and talents.

Racial equity means understanding the individual circumstances, environment, systems and experiences that have a disproportionately negative impact on an individual’s ability to function, participate and live their lives to their fullest potential because of their race; and then tailoring actions to eliminate those barriers. By doing so racial equality can be achieved, where opportunities, support and respect are equally afforded to everyone, and where efforts and achievements are seen and valued regardless of who the individual is or how they may present.

Another key concept that influenced the focus of the Framework was allyship. It was perceived to be instrumental in achieving racial competence. The meaning of allyship and lived experiences of REPAG related to allyship were continuously discussed during REPAG meetings, and REPAG members received training in allyship from members of Black African-, Asian- and Caribbean-heritage communities. The definition of allyship was adopted from NHS England and NHS Improvement [38]:

Allyship is about building relationships of trust, consistency and accountability with marginalised individuals and/or groups of people. Although you might not be a member of an under-invested or oppressed group, you can support them, make the effort to understand their struggle and use your voice alongside theirs.

A summary of key definitions used by the REPAG is provided in Supplementary File 1.

### Phase 2: developing an initial draft of the Framework (January 2021 – March 2021)

A plan with tasks and milestones for developing the Framework was agreed during the REPAG meeting on 26 January 2021. REPAG members from Black African-, Asian- and Caribbean-heritage communities (DS and FS) took the lead in reviewing the relevant literature and developing an initial draft of the Framework. Reflecting on their position in the process, the leads noted their unique position with public involvement in the NIHR to amplify the patient voice and facilitate public leadership (FS) and through work as a frontline clinician in the NHS (DF). A literature search (conducted by FS) indicated that while there were available tools for improving race equality in higher education [39] and in clinical trials [17], there was a lack of practical tools for improving racial equality in public involvement. A series of group discussions were held within the REPAG, where members brainstormed on potential areas of organisational activity and self-assessment questions. Through a combination of research expertise, organisational change experience, and lived experience, members created a wide range of suggested questions for organisations. The initial draft of the Framework contained key domains of organisational activity and corresponding self-assessment questions. These were based on the concepts and elements of good practice diagnosing and challenging systemic barriers to the under-representation of Black African-, Asian- and Caribbean-heritage communities in public engagement in the UK and internationally. The initial draft also contained some guidance on using the Framework. The initial draft was iteratively developed through meetings and discussions by all REPAG members over three months until April 2021. Importantly, REPAG public contributors identified the need for broader consultation with other public members.

### Phase 3: undertaking community consultations and modifying the draft (April 2021 – August 2021)

A total of 59 Black African-, Asian- and Caribbean-heritage participants (28 male and 31 female) were invited to three online community consultations via Zoom in May 2021 – June 2021, facilitated by REPAG Co-Chair (RJ). Black African- and Caribbean-heritage men were less likely to access health care than any other group of men or women; therefore, the first consultation event was specifically intended for Black African- and Caribbean-heritage men (20 attended). The other two consultation events were intended for participants of all genders who identified themselves as Black African-, Asian- and Caribbean-heritage (including mixed heritage). Recruitment to these events happened readily by REPAG public contributors sharing a promotional email through to individuals and community organisations with whom they have developed trusted relationships. This had the effect of widening the pool of NIHR public contributors, the majority of participants recruited (82%) had not worked with the NIHR previously, and 71% rated themselves as either having limited or some knowledge of health and care research. Public contributors registered to attend online. Accessibility and logistics needs were addressed as requested, including through the provisional of technical support during the meeting, and a recognition fee was provided to those who attended in line with NIHR policy [35].

Community consultations were divided into two parts: 1) general reflections about health and care research, and 2) specific reflections on the draft Framework. Written notes were taken, analysed thematically, and fed back to the REPAG. From the outset, REPAG Co-Chairs clarified that the event was not intended to be academic or professional, but a space where lived experiences and aspirations for change could be shared in a respectful yet meaningful and authentic way. The public Co-Chair (RJ) illustrated this by sharing a profoundly affecting aspect of their own experience. Anticipating the possibility that some attendees may be adversely affected by some of the topics raised, FS ensured that the onsite counsellor was available at every community consultation event.

A full report on the themes that emerged from the analysis of general reflections about health and care research is available open access elsewhere [40]. The key points from that analysis that informed the development of the Framework are summarised below [40]:

- Enduring issues of harm, betrayal, trauma and loss of confidence caused by racial injustices and cultural incompetencies must be acknowledged and respected.
- Researchers actively seeking to improve their cultural competencies must recognise and take responsibility for how to repair trust among communities.
- Community participants in research have neither been treated equitably, nor have derived value from the benefits of their involvement.
- Authentic, equal, open and transparent partnerships and relationships with community members must recognise contributions from and bring value to all contributors, and not continue to dehumanise or extract value from participants exploitatively.
- Diversity among and between communities is routinely ignored, misrepresented, or stereotyped leading to misdiagnosis.
- Cultural competency must be built on active consideration, understanding and respect of cultural differences.
- Community members experience collective ‘consultation fatigue’ and are sceptical of research consultations and ‘tick box’ exercises, with little meaningful action or outcomes that benefit the community consulted. Researchers should value diverse sources of knowledge and reduce Eurocentrism.
- We must learn from those failures and ensure that the wisdom of the multicultural community is acknowledged and the application of collective knowledge is our primary call to action.

Specific reflections on the draft Framework were reviewed and used by the REPAG to make modifications to the draft Framework. In line with the UK Standards for Public Involvement, participants were provided with the feedback on the aspects of their reflections, which were included in the modified version of the Framework, or a rationale for not including certain aspects (in the very few cases where these were out of the Framework’s scope). Key modifications made to the draft Framework in response to the community consultations are summarised below:

- Recommending to include members of the public/service users/research participants of diverse ethnic backgrounds as members of self-assessment teams
- Including health care professionals in the group with whom a self-assessment team is recommended to consult
- Recommending to include in the self-assessment process members of external organisations who can act as peer assessors
- Adding empathy as an attribute of a racially competent organisation
- Widening a number of questions on individual responsibility that include all staff
- Elaborating in the guide on the change management process to drive through the cultural change
- Checking the accessibility of the language and imagery before publication
- Revising the wording of questions to make them more personal
- Adding to all five domains SMART (specific, measurable, assignable, realistic and time-related) objectives with a view to ensuring that organisations hold themselves to account
- Revising the wording of questions to make them more precise and less open to interpretation

### Phase 4: piloting the framework (September 2021 – December 2021)

Sixteen organisations delivering health and care research in higher education, local government, the NHS, the private sector, and the voluntary sector piloted the Framework between September 2021 and December 2021. Pilot organisations were purposefully recruited to represent different types of academic and NHS partner organisations carrying out NIHR-funded research, as well as wider stakeholders from different sectors. The partner organisations had established relationships with NIHR public involvement and industry research collaboration staff involved in REPAG. Following the initial approach or expression of interest, FS and on occasions DF provided each partner organisation and individual briefing session on the framework and the pilot for a small team of mid to senior ranking representatives of the organisations. All the organisations agreed to proceed with the self-assessment following the briefing. A full list of pilot organisations is available on the NIHR website [41]. The purpose of the pilot was to test the feasibility of the self-assessment process as well as the usefulness and clarity of the questions.

Pilot organisations were requested to rate themselves on each of 60 self-assessment questions and provide narrative comments explaining a rationale and an evidence base for each rating. The recommended rating scale was: 0 = Not applicable to my organisation, 1 = Aspirational 'We want to be able to do this well', 2 = Emerging 'We're developing, but need more capability', 3 = Consolidating 'We do this well, but we are looking to improve', 4 = Transformational 'We do this really well and are open to sharing with others'. Pilot organisations were also recommended to carry out a consultation on the self-assessment questions with staff, clinicians, public contributors, patients, service users, carers and/or other members of the public in order to check self-ratings and if needed reassess them. Moreover, pilot organisations were requested to identify challenges, areas of good practice, potential actions, and consider providing an optional case study on actions taken, changes made and their benefits. Finally, pilot organisations were requested to rate the usefulness and clarity of each question on a scale from 1 to 5 and provide narrative comments on the questions with low scores (1 or 2).

In addition to submitting the requested materials to the REPAG, pilot organisations held an online “Share & Learn” event on 14 December 2021. The purpose of the event was to facilitate reflection by pilot organisations on their experiences of applying the Framework, and benefit from open discussion on progress, challenges, and learnings with REPAG members and five invited public contributors who previously participated in community consultations. The onsite counsellor was available for attendees for support during and after the meeting.

Throughout this phase, FS scheduled regular check-in sessions with the partner organisations and was on hand to answer questions. Identifying that the self-assessments and action plans of individual partner organisations were uncovering similar challenges; further facilitation was made available. This took the form of action learning sets [42]. The external trainer that contributed to the development of the REPAG training sessions, developed an action learning set facilitation course. Several action learning trained facilitators from the REF public contributors were trained as action learning facilitators, and then were supported in pairs to deliver joint sessions with two partner organisations. Common challenges were discussed, and next steps for overcoming barriers or addressing key decisions were explored. The public contributors were provided with recognition fees for their training and facilitation in line with NIHR policy [35].

### Phase 5: finalising the framework and accompanying materials (January 2022 – April 2022)

The materials submitted to the REPAG by pilot organisations were analysed and used to make modifications to the Framework. The results demonstrated the feasibility of conducting a meaningful self-assessment using the Framework over a period of three months and the usefulness of the results for developing longer-term action plans. Importantly, the scale to which each pilot partner organisations chose to apply their self-assessment and action plan varied from a contained department to a more strategic approach. For example, one partner organisation chose to apply the Framework to the development of a significant public facing applied health research project. It was observed that factors that affected the extent to which the partners were able to apply their self-assessment were the priority areas of work, capacity of internal staff, and public involvement resource allocation.

Key changes made to the Framework following the pilot were 1) reducing the number of self-assessment questions from 60 to 50 and 2) making the questions simpler and more focused all without losing the authenticity of the public input which formed these questions. The refined version of the Framework was then circulated to community consultation participants for their final comments and approval via email. REPAG public members were involved in all stages of the process.

The final version of the Framework is comprised of 50 self-assessment questions pertaining to the five domains of organisational activity: 1) individual responsibility, 2) leadership, 3) public partnerships, 4) recruitment, and 5) systems and processes. The five domains of the Framework are defined in Table 1. The self-assessment questions provide key concepts and elements of good practice that organisations should consider and address on their path to achieving racial competence. The final version of the self-assessment questions is provided in Supplementary File 2.

**Table 1:** The five domains of the NIHR Race Equality Framework defined

**Domain**	**Defined as**
**1. Individual responsibility**	Individuals being supported to take responsibility for highlighting, challenging and eliminating inequity, acting as allies to foster good relations.
**2. Leadership**	Behaviours that: a) Drive improvement. b) Empower individuals and harness their talents. c) Create a safe environment in which to challenge poor practice, where racial equity is prioritised, well-resourced and leads to tangible change.
**3. Public partnerships**	Equal partnerships that: a) Are respectful and provide a platform for learning and change. b) Instil a co-production ethos in all areas of work.
**4. Recruitment**	Implementing diversity recruitment strategies that: a) Use data to understand how and where to focus efforts to recruit Black African-, Asian- and Caribbean-heritage public contributors. b) Are monitored for their effectiveness.
**5. Systems and processes**	a) Identifying and removing barriers to involvement. b) Using flexible models and ways of working that recognise and respect the circumstances and experiences of Black African-, Asian- and Caribbean-heritage people.

The accompanying materials provide a guide to using the Framework with stages and steps, case studies, and allyship resources. The guide to using the Framework suggests 20 steps that organisations should take in three stages: 1) establishing organisational readiness, 2) carrying out a self-assessment, and 3) using results to improve racial competence. Case studies describe the implementation of the Framework during the pilot in seven different organisations representing NIHR research infrastructure, NHS, higher education, and industry, with examples of actions taken. Allyship resources include links to engaging talks and presentations explaining the concept of allyship, why it is important, and how to be a good ally.

In parallel, to support the diligent work and disseminate the Framework, a communication plan was developed by REPAG in consultation with NIHR in-house communications professionals. The communications professionals were supported by public contributors from REPAG, partner organisation representatives, and public contributors to the community consultation event, by providing media content for the online launch and sharing content with their networks.

### Availability

The Framework and accompanying materials were launched on 20 April 2022. In order to encourage its wide dissemination and adoption, it is available open access on the NIHR website in both HTML and accessible PDF formats with authentic visuals: https://www.nihr.ac.uk/documents/NIHR-race-equality-framework/30388.

## Discussion

In this article, we describe the process that was used to co-develop with public contributors and stakeholders the NIHR Race Equality Framework addressing five domains of organisational activity related to 1) individual responsibility, 2) leadership, 3) public partnerships, 4) recruitment, and 5) systems and processes. The framework has the potential to be transformative. It encourages organisations and individuals to do the necessary introspection (looking inwards at perceptions and attitudes) and self-reflection; (at one’s behaviours and actions) to understand the barriers which hinder meaningful participation and involvement. It is designed to focus attention on hearing, listening, and acting on the voices from marginalised communities, who are often unseen or unheard when discussions are held or decision are made in health and care research.

One of the most encouraging outcomes of this co-development process is the level of trust that it has engendered amongst the public contributors involved, many of whom have not previously been involved or engaged in NIHR funded and supported research. A significant number of the public contributors continue to partner with NIHR on various pieces of work beyond the Framework. Reflecting via a NIHR blog on their work, three public contributors agreed that “contributing to the Framework has been highly motivating and empowering and a great confidence-builder, not just for us personally but also in terms of inspiring us – as Asian women collectively, who have been victims of racial discrimination and treated unequally throughout our lives – to believe that racial equity in health and care services is possible” [43].

To the best of our knowledge, the Framework represents the first co-designed, organisation-wide self-assessment tool for addressing racial equality in public involvement in the UK and internationally. It adds to the growing body of patient and public involvement and engagement (PPIE) tools and methods. For example, the Framework adds to the UK Standards for Public Involvement [24], which defines the need for diversity of public involvement. The good practice guidelines for increasing the participation of Black, Asian and Minority Ethnic Communities in health and social care research [16], the INCLUDE Ethnicity Framework to improve inclusion of under-represented groups in clinical research studies [17], and the GRIPP2 reporting checklists for reporting patient and public involvement in research [44] focus on the research process and provide evidence-based recommendations to researchers and research teams. The Keele University Primary Care Research Centre’s model for the long-term sustainability of patient and public involvement focuses on institutional leadership and research infrastructure [18]. Our Framework contributes to this literature an organisation-wide approach to involving Black African-, Asian- and Caribbean-heritage communities in all aspects of research, by identifying and addressing specific areas of organisational activity which can contribute to improvements in racial equity in public involvement in research.

The Framework also informs public and government deliberations on research policy and strategy. For example, a government policy paper on the future of clinical research delivery mentions the Framework among the body of work contributing towards the government’s ambition for more people-centred research, i.e. “designed to make it easier for patients, service users and members of the public to access research of relevance to them and be involved in its design” [45]. An independent report for the Medical Research Council (MRC) considers the relevance of the Framework to supporting the development of a new MRC public involvement and engagement strategy [46]. A parliamentary report on Black maternal health mentions the Framework among the initiates that could contribute towards a better representation of Black women in maternal health research [47].

### Strengths

The process that was used to develop the Framework served to ensure its strengths with regard to co-production and co-design, authenticity, and rigour. Knowledge Mobilisation, in particular using a Community of Practice approach, helped to shape the development of the Framework. The Framework was co-produced with the public and potential users through the REPAG facilitated by REPAG public members and the NIHR, the UK’s largest public health and care research funding organisation. The membership of the REPAG represents public contributors, senior NIHR staff, and members of the health and academic communities, who all recognised the need to address racial equity in public involvement in research and had relevant knowledge and experience. The organisations that participated in the pilot represented different types of organisations delivering health and care research across different sectors, including higher education, local government, the NHS, the private sector, and the voluntary sector. Involvement of the NIHR in facilitating the process afforded public interest and relevance to practice across a wide range of academic-clinical partnerships carrying out NIHR-sponsored research for the benefit of diverse patient and public communities.

Another strength of the Framework is in the considerations of authenticity that were embedded throughout the process of the Framework development. Namely, the Framework, through the involvement of REPAG public contributors, was co-designed to reflect the cultural and lived experiences of the very communities it intends to support. The REPAG membership had an equal representation of non-white/white and female/male members. The key concepts underpinning the development of the Framework and the idea of the Framework were proposed by Black African-, Asian- and Caribbean-heritage members of the REPAG. During community consultations, 59 Black African-, Asian- and Caribbean-heritage participants were consulted on the initial draft of the Framework.

Yet another strength of the Framework is that it has been developed with rigour and due diligence. The UK Standards for Public Involvement were followed throughout the process of the Framework development. The GRIPP2 guidelines for reporting patient and public involvement in research were followed, and a relevant checklist is included in Supplementary File 3. The Framework is underpinned by the concepts of racial competence and allyship. The domains of organisational activity and corresponding self-assessment questions in the Framework are based on the concepts and elements of good practice in the UK and internationally. Feedback from community consultation was analysed thematically using established qualitative methods. The Framework was piloted among 16 organisations which represent the different types of academic and NHS partner organisations carrying out NIHR-funded research, as well as wider stakeholders from different sectors. Iterative changes to the Framework were made using deliberative and reflective practices. Finally, the Framework has provided timely practical advice for research organisations. In June 2020, the standardised Guidance for Applications used by NIHR’s research programmes introduced requirements that ‘equality, inclusion and diversity should also be properly considered when planning and describing the research and evidenced in the application’ [48]. Then in June 2021, the major overarching policy document for NIHR ‘Best Research for Best Health: The Next Chapter’ confirmed ‘embedding equality, diversity, and inclusion across NIHR’s research, systems and culture’ as one of the areas of its strategic focus, specifically stating that ‘NIHR is now systematically turning its attention to race and disability’ . In September 2022, NIHR published its ‘Equality, Diversity and Inclusion Strategy 2022-2027’ which ‘focused on reducing inequalities and maximising inclusion across our entire people framework which includes NIHR’s workforce, our research workforce, advisory workforce, research participants and the public’ [49]. The Framework provides guidance for those organisations who are seeking to address research inclusion in public involvement at a structural level with respect to race and health.

### Limitations

Despite its strengths, we recognise that the Framework has limitations. One limitation is that while early case studies show promise [36], at present, there are not yet any empirical data to demonstrate whether the adoption of the Framework will improve racial equality in public involvement and to what extent. The implementation of the Framework and evaluation of its effects is still in early stages and limited to the organisations that participated in the pilot. However, this is similar to the challenges when other equality tools were introduced, such as the Athena Swan Charter to improve gender equality and the INCLUDE Ethnicity Framework to improve inclusion of under-served groups in clinical research. Subsequent research has shown that the implementation of the Athena Swan Charter and the INCLUDE Ethnicity Framework has been associated with positive effects [50–52]. Therefore, we believe that the effects of the Framework are likely to be similar, provided each organisation commits to its implementation with sufficient resources and expertise.

Another limitation of the Framework is that it helps organisations to self-assess the current state, describes what a racially competent organisation looks like, and provides some guidance on how to develop an action plan without prescribing particular interventions. Developing and implementing an action plan with effective interventions requires organisational resources and expertise. Providing organisations with the dedicated resources is currently beyond the scope of the NIHR. It is expected that NIHR-sponsored organisations will resource the implementation of the Framework within the funding envelope provided for research infrastructure and PPIE in line with the requirements of the UK Equality Act 2010, the UK Standards for Public Involvement [24], and the NIHR Research Inclusion Strategy 2022-2027 [49]. Other organisations are encouraged to consider the implementation of the Framework as an investment to strengthen their organisational performance.

Yet another limitation of the Framework is that it does not provide guidance on addressing racial equality in an intersectional perspective, i.e. recognising that other individual protected characteristics, such as gender, class, ethnicity, sexuality, and others, can compound in conjunction with each other the experience of exclusion or discrimination [53, 54]. Research on gender and race in higher education shows that focusing on one protected characteristic could potentially have different effects on diversity and inclusion. On the one hand, prioritising one protected characteristic over other protected characteristics could lead to conflicting or “competing inequalities” agendas and action plans [55]. On the other hand, focusing agendas and action plans on one protected characteristic could have positive effects on diversity and inclusion across other protected characteristics as well [56, 57]. Those organisations that are interested in using the Framework should additionally consider how race intersects with other protected characteristics in their organisational context and plan actions accordingly.

Finally, given the variation in the scale and context that the partner organisations chose to apply their self-assessment to, we did not collect data on the amount of staff time and resources required to conduct the self-assessment. Instead, we included in the accompanying materials seven case studies demonstrating how different organisations conducted their self-assessment and formulated actions. We encourage the organisations that are interested in gauging the amount of time and effort for applying the Framework to consult the case studies and contact the relevant organisations.

### Transferability

Although the Framework focuses on Black African-, Asian- and Caribbean-heritage people and is primarily designed for organisations that involve patients and the public in health and care research in the UK, the questions of the Framework are likely to be relevant to other populations and settings. Because the Framework has been produced by a range of stakeholders, its principles may apply beyond the context of research organisations. The Framework can be potentially extended with some adaptions to any organisation or organisational unit that use partnerships with the public, e.g. in recruiting members of the public to its panels, committees or advisory groups, or involving members of the public as collaborators. Likewise, the Framework can be potentially extended with some adaptions to other groups with protected characteristics and under-represented communities. We are very interested in hearing from organisations in the UK and internationally who would like to try the Framework in different populations and settings.

### Future work

In our future work, we endeavour to promote the uptake and implementation of the Framework and capture and evaluate its impact. We encourage organisations that are interested in using the Framework to contact us so that we may offer our collaboration. Beyond the organisations that participated in the pilot, seven more organisations have adopted the Framework. As early adopters of the Framework implement interventions and action plans, we endeavour to evaluate the impact of the Framework on racial equality in public involvement and share the results. One of our pilot partners, Keele University has started work on the implementation of change in their PPIE practices based on the Framework with restructuring of their PPIE including the appointment of a Race Equality Ambassador for Public Involvement in Research. In addition to the learning resources on allyship and case studies included in the Framework, we endeavour to facilitate learning and training among interested organisations through “Share & Learn” events and online resources. Recently, we have published a new video of a poem that highlights the lived experiences of black men in the healthcare system [58]. We have also launched a Community of Practice Network, bringing together organisations who are dedicated to addressing racial inequities in health and care research and are committed to utilising the Race Equality Framework as a tool for driving cultural change. The Community of Practice Network has the following aims: 1) to create a safe environment for building a cohesive network for learning and sharing; 2) to facilitate opportunities for collaboration; and 3) to support the embedding of the Race Equality Framework in health and care research. Finally, we endeavour to regularly update the public, stakeholders, and potential collaborators on our work via the NIHR website and social media.

## Conclusion

The Framework represents the first co-designed self-assessment tool for addressing racial equality in public involvement in the UK and internationally. We encourage organisations in the field of health and care research and any other organisations that use partnerships with the public to consider adopting the Framework, and we offer our collaboration. Please send enquiries and suggestions torepag_enquiries@nihr.ac.uk.

### Supplementary Information

**Additional file 1. **Definitions.

**Additional file 2. **Self-assessment questions.

**Additional file 3. **GRIPP2 reporting checklist.

## Data Availability

All anonymised data generated or analysed during this study are included in this published article and its supplementary information files. Non-anonymised data generated or analysed during this study are not publicly available so that confidentiality can be maintained.

## Abbreviations

MRC: Medical Research Council
NIHR: National Institute for Health and Care Research
NHS: National Health Service
PPIE: patient and public involvement and engagement
REPAG: Race Equality Public Action Group
UK: United Kingdom
