# Supplementary material for: NIHR Race Equality Framework: development of a tool for addressing racial equality in public involvement
Source: Res Involv Engagem. 2024 May 7;10:44. doi: 10.1186/s40900-024-00569-z (PMC11077722; doi:10.1186/s40900-024-00569-z)
Supplement: Supplementary file 1 — Additional file 1. Definitions. [file 40900_2024_569_MOESM1_ESM.docx]

**NIHR Race Equality Framework for Public Involvement in Research definitions**

| **Term** | **Definition** |
| --- | --- |
| National Institute for Health and Care Excellence (NIHR), UK | NIHR aims to improve the health and wealth of the nation through research. The NIHR often works in collaboration with partners like charities, industry and the NHS to achieve outcomes. When the NIHR uses   - the word ‘research’ this encompasses health, public health and social care research; - the phrase ‘health and social care’, this includes public health; - the word 'public', this includes patients, people who draw on social care services, carers and communities.   https://www.nihr.ac.uk/ |
| NIHR Race Equality Public Action Group (REPAG) | REPAG was established in 2020. The group is co-chaired and led by public contributors of Black African, Asian and Caribbean heritage - each of whom brings their professional and lived experience - working alongside NIHR staff and members of the academic, health and care communities.  The purpose of the group is:   - to strengthen NIHR’s understanding of race inequality in health and social care research - to advise on actions for NIHR to take to ensure that race equality is embedded in the interactions between the research community and patients, people who use social care services, carers and the public - to prioritise the voices of those most affected by health and care challenges   https://www.nihr.ac.uk/about-us/our-key-priorities/equality-diversity-and-inclusion/race-equality-public-involvement.htm |
| Racial competence | Racial competence is the ability to recognise and check one's own bias; interact with racial diversity in a positive manner; and have open and honest conversations about race in ways that show a willingness to hear, learn and take action. Racial competence means understanding the impact of structural racism and fostering a culture of allyship that challenges organisational practices and behaviours that exclude Black African-, Asian and Caribbean-heritage people and other racialised groups. Being racially competent means translating our statements into action to promote equity of voice and equality of opportunity.  https://www.nihr.ac.uk/documents/nihr-race-equality-framework/30388 |
| Race | A race is a group of people defined by their colour, nationality (including citizenship) ethnicity or national origins. A racial group can be made up of more than one distinct racial group, such as Black British.  https://www.equalityhumanrights.com/equality/equality-act-2010/protected-characteristics |
| Racial equality | The Equality and Human Rights Commission (EHRC) describes equality as: ‘Ensuring that every individual has an equal opportunity to make the most of their lives and talents’. Racial equality means that opportunities, support and respect are equally afforded to everyone, and where efforts and achievements are seen and valued regardless of who the individual is or how they may present.  https://www.nihr.ac.uk/documents/nihr-race-equality-framework/30388 |
| Racial equity | Equity is about giving people what they need, in order to make things fair. Racial equity means understanding the individual circumstances, environment, systems and experiences that have a disproportionately negative impact on an individual’s ability to function, participate and live their lives to their fullest potential because of their race; and then tailoring actions to eliminate those barriers.  https://www.nihr.ac.uk/documents/nihr-race-equality-framework/30388 |
| Allyship | Allyship is about building relationships of trust, consistency and accountability with marginalised individuals and/or groups of people. Although you might not be a member of an under-invested or oppressed group, you can support them, make the effort to understand their struggle and use your voice alongside theirs.  https://www.england.nhs.uk/north-west/wp-content/uploads/sites/48/2021/05/A-guide-to-Allyship.pptx |
| Knowledge Mobilisation | Knowledge mobilisation strategies can help researchers share their research with the people who can use it. It is about sharing knowledge between different communities to create new knowledge to catalyse change. Knowledge mobilisation strategies should be integrated throughout your research programme.  Knowledge creation is seen as a process not a product.  It’s also known as knowledge translation, knowledge exchange or knowledge transfer.  https://www.nihr.ac.uk/researchers/i-need-help-designing-my-research/plan-knowledge-mobilisation.htm |
| Involvement | The NIHR defines public involvement in research as research being carried out ‘with’ or ‘by’ members of the public rather than ‘to’, ‘about’ or ‘for’ them.  https://www.nihr.ac.uk/ |
| Engagement | There is no standard model for engagement but it should demonstrate an approach that those in the community who are most affected are empowered to contribute towards decision-making.  https://www.nihr.ac.uk/ |
| Community Engagement and Involvement (CEI) | Key groups normally included in CEI are patients, carers and vulnerable groups. Community leaders, non-governmental and civil society organisations, faith groups, service commissioners and providers, and policy and law makers are examples of other types of stakeholders.  https://www.nihr.ac.uk/ |
| Public Involvement and Engagement (PPIE) | When NIHR uses the term ‘public’, they are including:   - patients and potential patients - carers and people who use health and social care services - people from organisations that represent people who use services.   When the public gets involved in research, they work alongside researchers to help shape:   - what research gets done - how it’s carried out - and how the results are shared and applied in practice.   Being involved is not the same as taking part in research. It's not about taking part in a trial or study to test a new treatment or care option. It’s about being a member of the research team that works together to design and run the study.  https://www.nihr.ac.uk/ |
| Community of Practice | A group of people, who share a concern, a set of problems, or a passion about a topic, and who deepen their knowledge and expertise in this area by interacting on an ongoing basis.  Wenger E, McDermott RA, Snyder W. Cultivating communities of practice: A guide to managing knowledge. Harvard Business Press; 2002. |
