# Supplementary material for: NIHR Race Equality Framework: development of a tool for addressing racial equality in public involvement
Source: Res Involv Engagem. 2024 May 7;10:44. doi: 10.1186/s40900-024-00569-z (PMC11077722; doi:10.1186/s40900-024-00569-z)
Supplement: Supplementary file 2 — Additional file 2. Self-assessment questions. [file 40900_2024_569_MOESM2_ESM.pdf]

DOMAIN 1

INDIVIDUAL RESPONSIBILITY

What to look for

Individuals are encouraged to reflect on how their attitudes and/or behaviours might disadvantage their Black African-, Asian- and Caribbean-heritage colleagues. They are challenged and supported to take responsibility for/seek opportunities to/set SMART goals for, enhancing their learning and development to improve their racial competence and their understanding of the impact of structural racism on health and care outcomes; and to build diverse networks beyond their own culture. Safe spaces for conversations about race are available, as is reverse mentoring and coaching on race. Teams understand what allyship is and how to be effective in this role. Black African-, Asian- and Caribbean-heritage public contributors are listened to, developed and supported.

Self-assessment questions

1. List all the race equality training (include training in allyship), that your organisation/ department provides (content, frequency, etc.). How does it ensure that all staff receive such training, and what efforts does it make to assess the quality/ impact of the training?

2. What further opportunities for learning and development in the area of race equality not listed in Q1 does your organisation/department make available to staff? (For example, reverse mentoring, coaching, building diverse networks that go beyond individuals' own culture.)

3. How does your organisation/department actively encourage staff to pursue race equality training, learning and development, as captured in the questions above? (For example, through individual performance objectives, setting SMART goals.)

4. How does your organisation/department measure the outcome of race-related training, learning and development in terms of the extent to which staff apply it in their work and day-to-day interactions with Black African-, Asian- and Caribbean-heritage people?

5. What is your organisation/department's definition of an 'ally'; and what form of allyship does it provide members of the public, as well as public contributors and members of staff of Black African, Asian or Caribbean heritage, in its everyday work and conversations? Give examples.

6. What support systems does your organisation/department have in place for Black African-, Asian- and Caribbean-heritage public contributors? (For example, a named contact whom they can approach on matters to do with race; a process for raising complaints to do with race.)

7. What development schemes does your organisation/department provide for Black African-, Asian- and Caribbean-heritage public contributors? (For example, mentoring, buddying scheme and/or shadowing opportunities.) How are they encouraged to use them and what is the uptake of such schemes?

8. Within your organisation/department, how are Black African-, Asian- and Caribbean-heritage public contributors and staff who experience everyday racism and/or micro-aggressions encouraged to challenge such behaviour?

9. How does your organisation/department support individuals to call out instances of discrimination or racism against Black African-, Asian- and Caribbean-heritage public contributors and staff, when they see it?

10. Within your organisation/department, how are individuals encouraged and supported to become advocates for Black African-, Asian- and Caribbean-heritage public contributors, staff and members of the public?

DOMAIN 2
LEADERSHIP

What to look for

Senior leaders are aware of the historical and current climate and context of race, as well as their responsibility to pursue solutions in this area. Strategies, plans and SMART goals are in place for engaging with and involving the public in work to improve organisational behaviours and practice. A senior leader executive champion provides visible leadership and accountability for driving organisational improvements and behavioural change. Senior leaders are open to and seek out opportunities for learning, such as reverse mentoring and/or coaching, to improve their racial competence. Initiatives to tackle racial inequity are fully resourced and supported; and examples of good practice are celebrated and shared.

Self-assessment questions

11. Does your organisation/department's vision for its health and care research strategy include becoming an anti-racist organisation? If so, how does this vision translate into organisational/departmental aims and objectives and ultimately into action? If not, how does your organisation/department make clear its commitment to racial equality in health and care research?
12. What steps have senior leaders taken to create an authentic, inclusive workplace environment that empowers and values the talents of Black African-, Asian- and Caribbean-heritage people?
13. What steps have senior leaders taken to encourage racial diversity on your organisation/department's advisory groups, committees, and patient and public involvement boards?
14. Do senior leaders consult the public directly on strategies and policies to do with race? If yes, what examples are there of such consultation being used to shape strategy and policy in your health and care research?
15. Has your organisation/department appointed any senior champions from its executive team to lead on matters of race? (For example, as the lead on racial equity/equality in health and care research, or as a freedom-to-speak-up guardian or advocate.)
16. How have senior leaders in your organisation/department demonstrated their commitment to the Race Equality Framework? (For example, how and to whom they have allocated this work, the strategies and resources they have put in place to support them and how they intend to review the process and outcomes.)
17. What measures does your organisation/department have in place to ensure that it is open about the number and outcome of complaints it receives against managers and senior leaders where racism and/or discrimination appears to be a factor?
18. How do senior leaders in your organisation/department ensure that the research cycle does not contribute to racial bias and that any bias that is found is eliminated?
19. How do senior leaders promote the value and importance of building partnerships with the public?
20. What training, mentoring and coaching on racial equality/equity and anti-racism is provided for senior leaders; and what checks are in place to ensure that everyone eligible receives it?

DOMAIN 3

PUBLIC PARTNERSHIPS

What to look for

Surveys and audits demonstrate efforts to partner with Black African-, Asian- and Caribbean-heritage people and communities. Black African-, Asian- and Caribbean-heritage people are actively involved in co-production work and in informing and shaping priorities. Relationships are established and sustained through continued engagement and dialogue. Black African-, Asian- and Caribbean-heritage people report positive experience of their interactions and colleagues are appreciative and recognise and value the contributions they make.

Self-assessment questions

21. What action has your organisation/department taken, or what evidence has it used, to understand how Black African-, Asian- and Caribbean-heritage communities view their relationship with the healthcare system? (For example, lack of trust, experience of discrimination.)

22. How does your organisation/department internally encourage the involvement of Black African-, Asian- and Caribbean-heritage communities in its work?

23. How does your organisation/department go about identifying which communities it is not engaging with?

24. Does your organisation/department require research leaders to establish long-term links with Black African-, Asian- and Caribbean-heritage community groups to help build the latter's trust and confidence? If so, how does it go about this? What benefits to both the organisation and those communities have those links brought?

25. What partnerships has your organisation/department built with organisations or individuals of Black African, Asian and Caribbean heritage with a view to:
    a) being able to inform them of opportunities to participate in research
    b) understanding barriers to their participation in research
    c) co-designing research, and
    d) them informing and shaping research priorities?

26. Does your organisation/department ever sub-contract/contract Black African-, Asian- or Caribbean-heritage organisations to carry out research? If not, does it have any intentions to do so? If not, please give reasons for your answer.

27. What opportunities and incentives does your organisation/department offer Black African-, Asian- and Caribbean-heritage public contributors to apply their skills, lived experiences and diversity to other activities not related to research—in particular, to inform or lead on training on race or to sit on selection/interview panels?

28. How does your organisation/department define and measure improvement in terms of public partnership activities? (For example, number or type of partnerships, diversity profile, outcomes achieved.) How does it recognise and share progress and good practice in this area?

29. How does your organisation/department monitor how the people with whom it engages, as public contributors, research participants or panel/committee/board members, view their interactions? (For example, the extent to which they feel heard and their contributions are valued by the healthcare system.) Can the results be analysed by race?

30. How does your organisation/department communicate its appreciation to public contributors and make them feel recognised and valued for their contributions?

DOMAIN 4

RECRUITMENT

What to look for

Relationships are established with community groups/organisations to build trust and understand barriers to recruitment. Strategies are based on evidence and best practice. SMART recruitment targets are set and monitored, effectiveness is reviewed and action is taken to tackle shortcomings. Diverse media are used. Information is tailored and culturally sensitive in terms of language. Partnering with Black African-, Asian- and Caribbean-heritage organisations forms a key part of organisational/departmental recruitment efforts.

Self-assessment questions

31. How does your organisation/department use data to understand the demographic profile of local areas, and how does it use this information to inform where to focus its recruitment activities?
32. How does your organisation/department review its recruitment and selection processes to determine whether they are delivering the organisation/department's desired racial equity outcomes?
33. What does your organisation/department do to ensure that its recruitment and selection processes (for public contributors, research participants and panel/board/committee members) are not biased against/do not disadvantage people of Black African, Asian or Caribbean heritage?
34. What does your organisation/department do to ensure that Black African-, Asian- or Caribbean-heritage communities know about opportunities to become public contributors, research participants and panel/board/committee members? (For example, through long-term relationships with patients, service users and carers, information sessions in local venues, outreach work, articles in community newspapers or other diverse media.)
35. With regard to Q34 above, how does your organisation/department decide what action to take in order to reach communities?
36. What attempts has your organisation/department made to understand the reasons behind Black African-, Asian- and Caribbean-heritage people not applying for positions as public contributors, research participants or panel/board/committee members? (For example, mistrust of research aims, fear of tokenism.)
37. With regard to Q36 above, what actions has your organisation/department taken to challenge those reasons and how successful have those actions been?
38. What attempts does your organisation/department make to encourage applications from Black African-, Asian- and Caribbean-heritage people through its recruitment materials? (For example, materials are published in various languages, contain diverse images, include a statement expressing the desire to recruit from these communities.) How successful have these attempts been?
39. If not covered in the questions above, what attempts has your organisation/department made to apply recognised best practice to how it recruits Black African-, Asian- and Caribbean-heritage public contributors, research participants or panel/board/committee members? How successful have these attempts been?
40. Does your organisation/department have a budget for recruiting public contributors (of all backgrounds); if so, what is the process for setting and reviewing it?

DOMAIN 5

SYSTEMS & PROCESSES

What to look for

Strategies for public involvement set out clear plans and SMART goals for addressing the under-representation of Black African-, Asian- and Caribbean-heritage public contributors. Public-facing policies on health and care research are impact-assessed and there is evidence of learning from Black African-, Asian- and Caribbean-heritage communities. There are flexible models and ways of working that speak to the circumstances of Black African-, Asian- and Caribbean-heritage people.

Self-assessment questions

41. What systems/processes does your organisation/department have in place to help it understand the health and care needs of the population it serves? How comprehensive is the information they provide?

42. How regularly does your organisation/department carry out race equality impact assessments on its public-facing policies and projects on health and care research? What processes are in place to ensure that actions falling out of these assessments are carried out in a timely manner?

43. How often does your organisation/department audit its systems and processes to identify barriers to the public involvement of Black African-, Asian- and Caribbean-heritage people? (For example, the language in which material is written, the use of digital communication tools.) What processes are in place to ensure that actions identified by these audits are carried out in a timely manner?

44. What models or ways of working has your organisation/department introduced that acknowledge the circumstances of Black African-, Asian- and Caribbean-heritage people? (For example, outreach activity at weekends.)

45. What is your organisation/department's approach to co-producing research with Black African-, Asian- and Caribbean-heritage people? How does it ensure that its approach is effective?

46. Within your public involvement work, what systems/processes does your organisation/department have in place to monitor the racial diversity profile of public contributors, research participants and panel/board/committee members?

47. With regard to Q46 above, how does your organisation/department then use this information? (For example, to identify under- or over-representation amongst certain groups; to set targets for maintaining or increasing representation in certain groups.)

48. What is your organisation/department's process for deciding what actions it will take in response to suggested changes, benefits and learning identified through Black African-, Asian- and Caribbean-heritage public involvement?

49. How does your organisation/department record all the learning outcomes (whether favourable or critical) from its public involvement activities and then share these with staff and members of the public?

50. How does your organisation/department encourage its funded partners (including research collaborators, contractors and other third parties) to demonstrate racial equity in terms of awarding research contracts to Black African-, Asian- and Caribbean-heritage people or to organisations that represent them?
