# Supplementary material for: NIHR Race Equality Framework: development of a tool for addressing racial equality in public involvement
Source: Res Involv Engagem. 2024 May 7;10:44. doi: 10.1186/s40900-024-00569-z (PMC11077722; doi:10.1186/s40900-024-00569-z)
Supplement: Supplementary file 3 — Additional file 3. GRIPP2 reporting checklist. [file 40900_2024_569_MOESM3_ESM.docx]

**GRIPP2 long form**

| **Section and topic** | **Item** | **Reported on page No** |
| --- | --- | --- |
| Section 1: Abstract of paper | | |
| 1a: Aim | Report the aim of the study | Page 2  This paper describes how the UK’s National Institute for Health and Care Research (NIHR) convened a Race Equality Public Action Group (REPAG), which co-developed with public contributors and stakeholders a Race Equality Framework to help organisations improve racial equality in public involvement. |
| 1b: Methods | Describe the methods used by which patients and the public were involved | Page 2  The REPAG, through meetings and discussions, defined the focus of the Framework, and public contributor (DF) and REPAG Co-chair (FS) developed an initial draft of the Framework. Public contributors identified the need for broader consultation with other public members. Three community consultation events with a total of 59 members of Black African-, Asian- and Caribbean-heritage communities were held to seek their views on health and care research generally and on the draft Framework specifically. The draft Framework was modified and piloted among 16 organisations delivering health and care research. Following feedback from the pilot, the Framework was modified and prepared for publication. |
| 1c: Results | Report the impacts and outcomes of PPI in the study | Page 2  The Framework is designed as a self-assessment tool comprised of 50 questions pertaining to five domains of organisational activity: 1) individual responsibility, 2) leadership, 3) public partnerships, 4) recruitment, and 5) systems and processes. The questions were co-designed with REPAG public members and provide key concepts and elements of good practice that organisations should consider and address on their path to achieving racial competence. |
| 1d:Conclusions | Summarise the main conclusions of the study | Page 2  The Framework represents the first self-assessment tool for improving racial equality in public involvement. Co-design with REPAG public members enhanced its authenticity and practicality. Organisations in the field of health and care research and any other organisations that use partnerships with the public are encouraged to adopt the Framework. |
| 1e: Keywords | Include PPI, “patient and public involvement,” or alternative terms as keywords | Page 3  Race equality, antiracism, inequality, diversity, inclusion, public involvement, public engagement, health research, NIHR |
| Section 2: Background to paper | | |
| 2a: Definition | Report the definition of PPI used in the study and how it links to comparable studies | Page 4  Public involvement in research entails that members of the public are actively involved in research projects and in research organisations to the effect that “research being carried out ‘with’ or ‘by’ members of the public rather than ‘to’, ‘about’ or ‘for’ them” [20]. Public members can offer lived experience (expertise by experience). |
| 2b: Theoretical underpinnings | Report the theoretical rationale and any theoretical influences relating to PPI in the study |  |
| 2c: Concepts and theory development | Report any conceptual models or influences used in the study | Page 11  The UK Standards for Public Involvement were followed throughout the process of the Framework development. The GRIPP2 guidelines for reporting patient and public involvement in research were followed, and a relevant checklist is included in Supplementary File 2. The Framework is underpinned by the concepts of racial competence and allyship. The domains of organisational activity and corresponding self-assessment questions in the Framework are based on the concepts and elements of good practice in the UK and internationally. Feedback from community consultation was analysed thematically using established qualitative methods. The Framework was piloted among 16 organisations which represent the different types of academic and NHS partner organisations carrying out NIHR-funded research, as well as wider stakeholders from different sectors. Iterative changes to the Framework were made using deliberative and reflective practices. |
| Section 3: Aims of paper | | |
| 3: Aim | Report the aim of the study | Page 4  To provide focused guidance to practitioners of public involvement in health and care research the REPAG decided to co-develop with public contributors and stakeholders the NIHR Race Equality Framework – a self-assessment tool to help organisations improve racial equality in public involvement. Below, we describe the process that was used by the REPAG to develop and launch the Framework. |
| Section 4: Methods of paper | | |
| 4a: Design | Provide a clear description of methods by which patients and the public were involved | Page 6  Between October 2020 and April 2022, REPAG members held monthly meetings, typically two hours long, and facilitated most of the time online via Zoom, with both plenary and break out group sessions. In order to provide a strong foundation for this work by preparing REPAG members for honest and reflective conversations, FS recruited an external trainer with significant experience in this area. With NIHR occupying a strategic position within the health and care research landscape, the group discussed recommendations it could disseminate to research active organisations that it funds, that they could address the documented barriers to:  • Meaningful involvement of public contributors from communities under-served by research [17];  • Co-production and testing of interventions shaped to address different support needs and tackle known disparities in health and care outcomes [33], in line with UK public sector equality duties [34].  Ideas and suggestions were captured and aggregated using Jamboard, a digital interactive whiteboard, as well as via email. Key to the success of these meetings was the conscious steps taken and efforts made to ensure trust, confidence, a safe space for REPAG deliberations, and equal partnerships with the REPAG public contributors. |
| 4b: People involved | Provide a description of patients, carers, and the public involved with the PPI activity in the study | Page 6  A full list of the 19 people who have made a substantial contribution to the Framework in one or more phases is given in the acknowledgements. They were past and present REPAG members representing the NIHR (9), NIHR and REPAG public contributors (6), and academic partners (4). REPAG public contributors were recruited from existing public involvement networks known to the NIHR Senior Public Involvement Manager and REPAG Founder and Co-Chair (FS). |
| 4c: Stages of involvement | Report on how PPI is used at different stages of the study | Page 6  REPAG Public members were involved in five major phases of work:   1. Emergence of the idea of the Framework 2. Developing an initial draft of the Framework 3. Undertaking community consultations and modifying the draft 4. Piloting the Framework 5. Finalising the Framework and accompanying materials |
| 4d: Level or nature of involvement | Report the level or nature of PPI used at various stages of the study | Page 6  REPAG public members were involved in all stages of the process.  Page 9  A total of 59 Black African-, Asian- and Caribbean-heritage participants (28 male and 31 female) were invited to three online community consultations |
| Section 5: Capture or measurement of PPI impact | | |
| 5a: Qualitative evidence of impact | If applicable, report the methods used to qualitatively explore the impact of PPI in the study |  |
| 5b: Quantitative evidence of impact | If applicable, report the methods used to quantitatively measure or assess the impact of PPI |  |
| 5c: Robustness of measure | If applicable, report the rigour of the method used to capture or measure the impact of PPI |  |
| Section 6: Economic assessment | | |
| 6: Economic assessment | If applicable, report the method used for an economic assessment of PPI |  |
| Section 7: Study results | | |
| 7a: Outcomes of PPI | Report the results of PPI in the study, including both positive and negative outcomes | Page 9  A total of 59 Black African-, Asian- and Caribbean-heritage participants (28 male and 31 female) were invited to three online community consultations via Zoom in May 2021 – June 2021, facilitated by REPAG Co-chair (RJ). In order to widen the pool of NIHR public contributors, the majority of participants (82%) were recruited among those who had not worked with the NIHR previously, and 71% rated themselves as either having limited or some knowledge of health and care research. Community consultations were divided into two parts: 1) general reflections about health and care research, and 2) specific reflections on the draft Framework. Written notes were taken, analysed thematically, and fed back to the REPAG.  Specific reflections on the draft Framework were reviewed and used by the REPAG to make modifications to the draft Framework. In line with the UK Standards for Public Involvement, participants were provided with the feedback on the aspects of their reflections, which were included in the modified version of the Framework, or a rationale for not including certain aspects (in the very few cases where these were out of the Framework’s scope). |
| 7b: Impacts of PPI | Report the positive and negative impacts that PPI has had on the research, the individuals involved (including patients and researchers), and wider impacts |  |
| 7c: Context of PPI | Report the influence of any contextual factors that enabled or hindered the process or impact of PPI |  |
| 7d: Process of PPI | Report the influence of any process factors, that enabled or hindered the impact of PPI |  |
| 7ei: Theory development | Report any conceptual or theoretical development in PPI that have emerged |  |
| 7eii: Theory development | Report evaluation of theoretical models, if any |  |
| 7f: Measurement | If applicable, report all aspects of instrument development and testing (eg, validity, reliability, feasibility, acceptability, responsiveness, interpretability, appropriateness, precision) |  |
| 7g: Economic assessment | Report any information on the costs or benefit of PPI |  |
| Section 8: Discussion and conclusions | | |
| 8a: Outcomes | Comment on how PPI influenced the study overall. Describe positive and negative effects | Page 14  The Framework was co-produced with the public and potential users through the REPAG facilitated by REPAG public members and the NIHR, the UK’s largest public health and care research funding organisation.  Another strength of the Framework is in the considerations of authenticity that were embedded throughout the process of the Framework development. Namely, the Framework, through the involvement of REPAG public contributors, was co-designed to reflect the cultural and lived experiences of the very communities it intends to support. The REPAG membership had an equal representation of non-white/white and female/male members. The key concepts underpinning the development of the Framework and the idea of the Framework were proposed by Black African-, Asian- and Caribbean-heritage members of the REPAG. During community consultations, 59 Black African-, Asian- and Caribbean-heritage participants were consulted on the initial draft of the Framework. |
| 8b: Impacts | Comment on the different impacts of PPI identified in this study and how they contribute to new knowledge |  |
| 8c: Definition | Comment on the definition of PPI used (reported in the Background section) and whether or not you would suggest any changes |  |
| 8d: Theoretical underpinnings | Comment on any way your study adds to the theoretical development of PPI |  |
| 8e: Context | Comment on how context factors influenced PPI in the study |  |
| 8f: Process | Comment on how process factors influenced PPI in the study | Page 13  One of the most encouraging outcomes of this co-development process is the level of trust that it has engendered amongst the public contributors involved, many of whom have not previously been involved or engaged in NIHR funded and supported research. A significant number of the public contributors continue to partner with NIHR on various pieces of work beyond the Framework. Reflecting via a NIHR blog on their work, three public contributors agreed that “contributing to the Framework has been highly motivating and empowering and a great confidence-builder, not just for us personally but also in terms of inspiring us – as Asian women collectively, who have been victims of racial discrimination and treated unequally throughout our lives – to believe that racial equity in health and care services is possible” [43]. |
| 8g: Measurement and capture of PPI impact | If applicable, comment on how well PPI impact was evaluated or measured in the study |  |
| 8h: Economic assessment | If applicable, discuss any aspects of the economic cost or benefit of PPI, particularly any suggestions for future economic modelling. |  |
| 8i: Reflections/critical perspective | Comment critically on the study, reflecting on the things that went well and those that did not, so that others can learn from this study |  |

PPI=patient and public involvement
